# Integrated analysis of transcriptomic data reveals the platelet response in COVID-19 disease

**DOI:** 10.1038/s41598-022-10516-1

**Published:** 2022-04-27

**Authors:** Leonardo D. Garma, Hansen Deng, Ezequiel Goldschmidt

**Affiliations:** 1 Department of Medical Biochemistry and Biophysics, Karolinska Institutet, Stockholm, Sweden; 2 Department of Neurosurgery, University of Pittsburgh Medical Center, Pittsburgh, PA USA; 3 Department of Neurosurgery, University of California San Francisco, San Francisco, CA USA

**Keywords:** Pathogenesis, Infection, Viral infection, Thrombosis

## Abstract

COVID-19 is associated with an increased risk of thrombotic events. However, the pathogenesis of these complications is unclear and reports on platelet infection and activation by the virus are conflicting. Here, we integrated single-cell transcriptomic data to elucidate whether platelet activation is a specific response to SARS-CoV-2 infection or a consequence of a generalized inflammatory state. Although platelets from patients infected with SARS-CoV-2 over expressed genes involved in activation and aggregation when compared to healthy controls; those differences disappeared when the comparison was made with patients with generalized inflammatory conditions of other etiology than COVID-19. The membrane receptor for the virus, ACE-2, was not expressed by infected or control platelets. Our results suggest that platelet activation in patients with severe COVID-19 is mainly a consequence of a systemic inflammatory state than direct invasion and activation.

## Introduction

Life threatening coronavirus disease 2019 (COVID-19) results from the combination of severe pneumonia and a variety of extrapulmonary complications; amongst them, thrombotic events contribute to disease progression and organ damage. While part of the disseminated intravascular coagulopathy spectrum, thrombotic incidents are overrepresented in patients with COVID-19, resulting in an abnormally high rate of myocardial infarctions, strokes and embolic events^1,2^.

Platelets participate both in hemostasis regulation and antiviral response, and reports on their participation in the pathogenesis of COVID-19 related thrombosis are mixed^3–5^. According to most authors, platelets lack the membrane receptor for SARS-CoV-2 ACE2, and thus, are not susceptible to direct viral invasion and cytotoxicity^6^, although two recent studies suggest that the virus does infect platelets via an ACE2-independent mechanism^7,8^. Platelets participate in the innate immune antiviral response mainly via toll-like, NOD-like and C-type lectin receptors, and increased platelet activation has been reported in patients with COVID-19^9^. Consistently platelet activation is seen in a wide array of lung inflammatory conditions^10,11^.

Recently, Banne et al. compared bulk sequencing of ten patients infected with Covid-19 to five heathy donors^6^ finding increased expression of genes related to antigen presentation, mitochondrial dysfunction, protein ubiquitination and platelet activation. This study strongly argues in favor of a platelet role in the pathogenesis of COVID-19 related thrombosis; nevertheless, the comparison with healthy donors and the small sample can limit the scope of these findings^12^. Koupenova et al. found that platelets do internalize virions and that this leads to the upregulation of apoptotic and necroptotic pathways, rather than platelet activation or pro-thrombotic pathways^8^.

Lately, several authors reported on peripheral blood single-cell RNA sequencing (scRNA-seq) corresponding to healthy individuals and a number of disease conditions, including COVID-19^13–18^. Although platelets usually receive little to no attention, the platelet transcriptome is available in these reports, allowing us to compare their gene expression in different conditions. By doing so, we were able to increase our sample size, and discern a SARS-Cov-2 specific platelet response from platelet activation derived from a non-specific generalized inflammatory response.

## Results

### Data collection, preprocessing and integration

We used three peripheral blood mononuclear cells scRNA-seq datasets^13,17,18^ from healthy patients (22), patients with COVID-19 (39) as well as patients with acute lung inflammatory conditions non related to SARS-CoV-2 (11).

**Figure 1:**
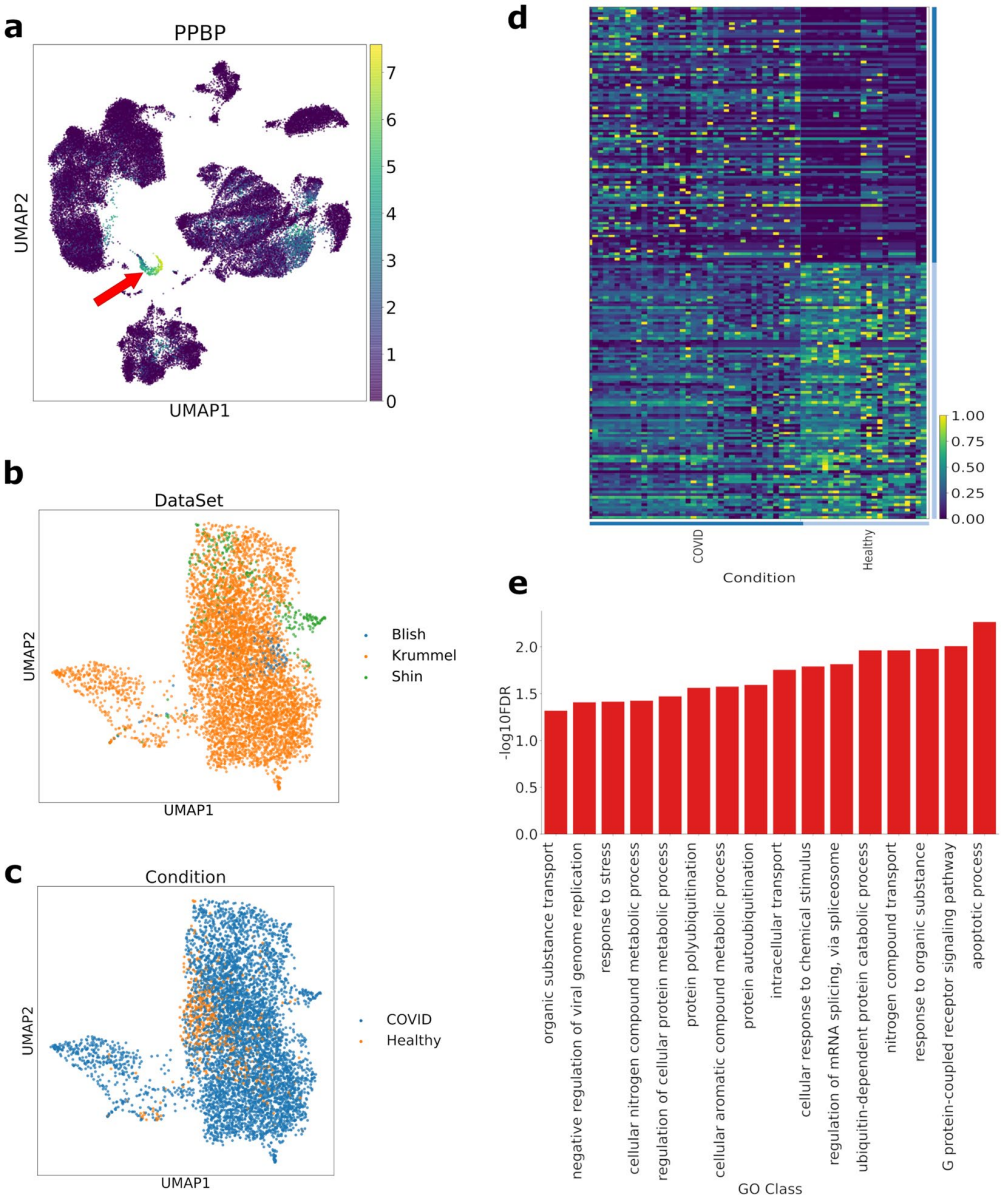
Integration of three single cell datasets and overview of the transcriptomic changes in platelets from COVID-19 patients. (**a**) UMAP projection of the cells in the dataset from Lee et al.^18^ colored by their normalized expression of the platelet marker gene PPBP. The platelets form an easily identifiable cluster separated from the rest of the cell types, indicated by the red arrow marker. (**b**) UMAP projection of the platelets from the three combined datasets after mutual nearest neighbors integration. The cells are colored by origin, showing that there is no obvious separation by dataset. The inter-dataset distances are larger than the distances between cells of the same dataset. (**c**) UMAP projection of the platelets colored by condition. (**d**) Heatmap showing the normalized expression levels of the top 100 most differentially expressed genes between healthy subjects and COVID-19 patients, summarized by subject. Biological process classes enriched in the set of upregulated genes in the platelets of COVID-19 patients. The terms are sorted by the significance of their enrichment, assessed by their false discovery rates.

We applied quality control filters and normalization to each of the three datasets independently (see “Methods”). The filtered and normalized data was clustered, and platelets were identified based on known markers (PBPP, TUBB1 and LIMS1). Figure 1a illustrates how 300 cells were identified as platelets in the dataset of Lee et al.^18^. We then integrated the data using mutual nearest neighbors batch correction^19^, in order to visualize the differences by various sample datasets and by COVID-19 status (Fig. 1b,c).

### ACE2 receptor and Covid-19 genome

There were no counts assigned to the Covid-19 receptor ACE2 in the platelet cells extracted from any of the three datasets. Only the dataset from Wilk et al.^13^ had used a Covid-19 reference genome to align the sequencing data, and in this case, no read was aligned to any of the known viral genes.

### Transcriptomic differences between COVID-19 patients and control subjects

In order to compare the differences in gene expression across conditions, we performed a differential expression analysis using a pseudo-bulk approach, which has been shown to outperform other methods when comparing individuals across conditions^20^. Comparing the COVID-19 patients and control subjects, our analysis found broad differences in the transcriptome of the subjects across conditions, with a total of 373 significantly upregulated and 51 significantly downregulated genes (Supplementary Table 1). These differences in expression are illustrated in Fig. 1d.

A number of biological processes appeared to be enriched in the ensemble of upregulated genes (Fig. 1e**,** Supplementary Table 2). Notably, these included apoptosis as the most significantly enriched process, and ubiquitination and stress responses, but no platelet activation or pro-thrombotic pathways.

**Figure 2:**
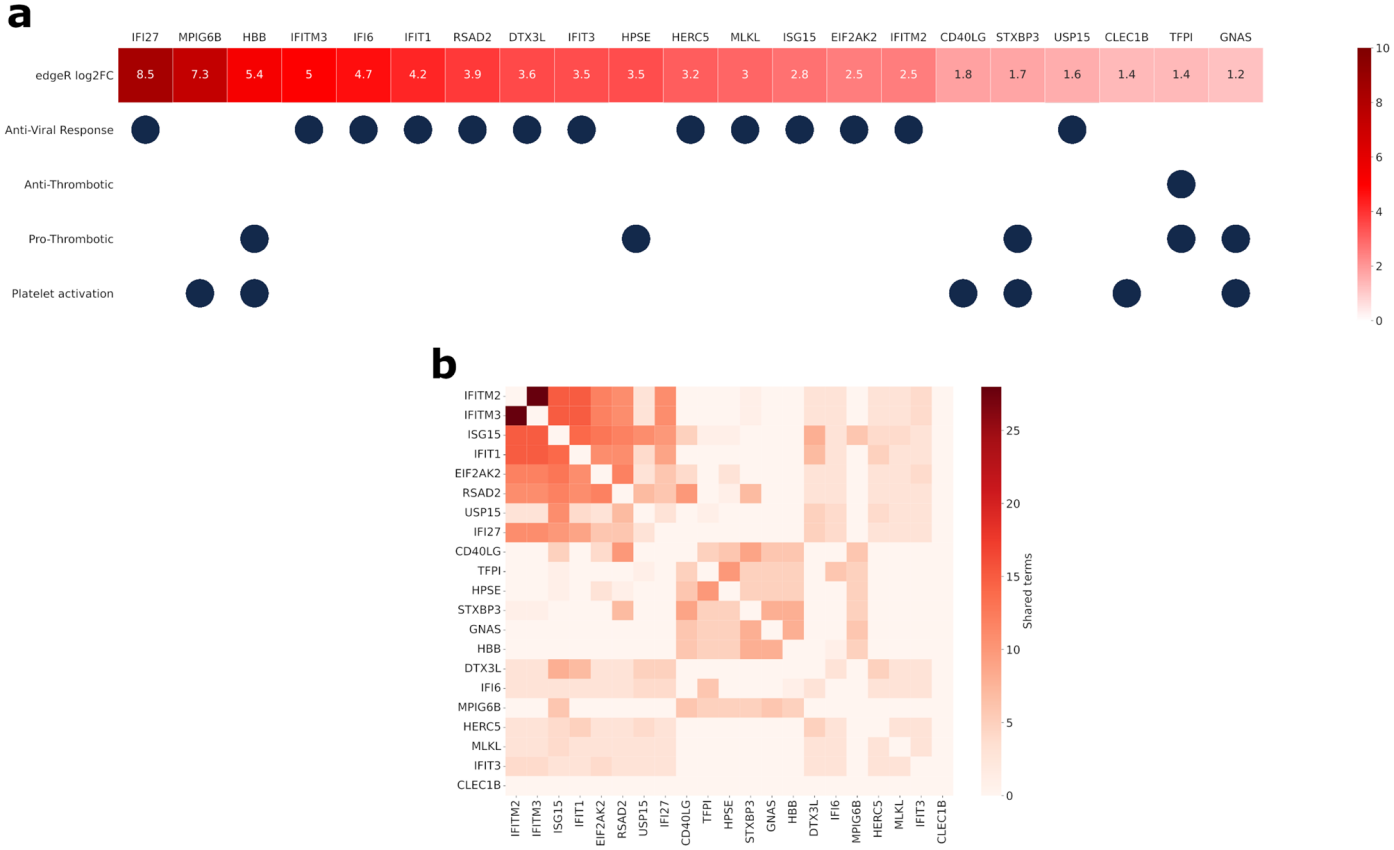
(**a**) Anti-viral, thrombotic-related and platelet activation genes with significantly different expression levels between healthy subjects and COVID-19 patients. The top row indicates the log_2_ fold change values, whereas the GO-terms related to each gene are indicated below. (**b**) Enriched GO terms (biological process) terms shared by each pair of the genes in (**a**).

Among the genes with significant differences in expression, we investigated the influence of SARS-CoV-2 infection on three specific processes: i) thrombosis, ii) platelet activation and iii) anti-viral response. In total, we detected 21 genes related to these processes with a significant difference in expression between healthy controls and COVID-19 patients (Fig. 2).

Eight genes associated with platelet activation and pro-thrombosis were upregulated in COVID-19 patients: MPIG6B, HBB, HPSE, CD40LG, STXBP3, CLEC1B, TFPI and GNAS. *MPIG6B* (megakaryocyte and platelet inhibitory receptor G6b) gene encodes for a plasma membrane-bound cell surface receptor. It is a critical regulator of hematopoietic differentiation, megakaryocyte and platelet production. *HBB* (hemoglobin subunit beta) gene, encodes for the beta-globin subunit of hemoglobin located in red blood cells. HPSE (heparinase) is an enzyme that cleaves heparan sulfate proteoglycans and releases bioactive molecules from the extracellular matrix, which in turn act as pro-thrombotic by increasing the factors VII and X. CD40LG stimulates T cells and STXBP3 selectively responds to elevated calcium in platelets. *CLEC1B* (C-type lectin domain family 1 member B) encodes for receptor that facilitates platelet aggregation; TFPI (Tissue Pathway Factor Inhibitor) is part of an autoregulatory loop that inhibit proteases for factor X and VIIa. GNAS (GNAS complex locus) is a complex imprinted locus encoding for G proteins that functions as transducers in various signaling pathways.

We found 13 genes that were associated with anti-viral response, including the interferon induced proteins IFI27, IFITM3, IFI6, IFIT1, IFIT3 and IFITM2, which are part of the innate antiviral response. *IFI27* (interferon alpha inducible protein 27) gene is involved in Interferon alpha/beta and gamma signaling pathways, including induced apoptosis and transcriptional inhibition. *IFITM3 (*interferon induced transmembrane protein 3) gene encodes for antiviral protein that disrupts intracellular cholesterol homeostasis and inactivates new enveloped viruses. *IFI6* (interferon alpha inducible protein 6) gene plays a role in regulating apoptosis and has antiviral activity through inhibition of viral entry into cells. *IFITM2* (interferon induced transmembrane protein 2) encodes for an antiviral protein that inhibits viral entry and release of viral content while facilitates cellular endocytosis.

We investigated the relationships amongst the 21 genes by examining their common biological processes. A GO Enrichment analysis revealed that the set of genes was significantly enriched in 571 different biological processes (Supplementary Table 7). We computed the number of common terms between each pair of genes in the set to reveal the connectivity within the ensemble. This analysis indicates that the viral-response genes and the genes related to thrombosis and platelet activation form two clearly distinctive communities, with few shared links between them (Fig. 2b).

### Transcriptomic differences between COVID-19 and other acute respiratory conditions

We hypothesized that platelet expression changes seen in COVID-19 patients is also be observed in patients with other acute respiratory infections, instead of being SARS-CoV-2 specific. An adequate control sample to test this hypothesis would be patients with severe lung and systemic inflammatory conditions unrelated to SARS-CoV-2. We utilized the data generated by Combes et al. which had a sample of COVID-patients and patients diagnosed with acute respiratory conditions unrelated to the virus^17^.

In this sample, we found that most of the genes did not vary by expression level. The list of genes with significant changes in expression was reduced to 39, with 33 upregulated genes and 6 downregulated ones (Supplementary Table 3). According to our analysis, neither the upregulated nor the downregulated ensembles were significantly enriched in any specific biological processes.

**Figure 3:**
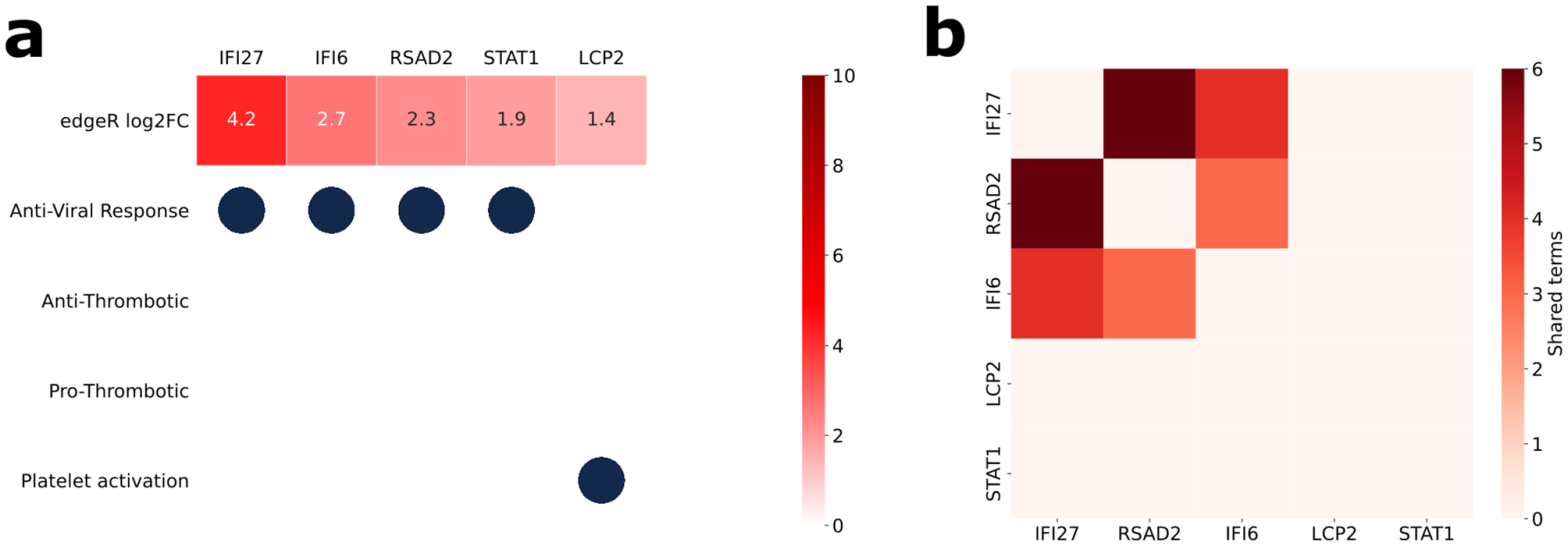
(**a**) Genes with differential expression between COVID-19 patients and subjects with other lung inflammatory conditions related to the selected GO-terms. The log_2_ fold change values are indicated in the top row and the GO terms annotated for each gene are shown below. (**b**) Matrix representation of the number of enriched GO terms shared by each pair of genes in (**a**).

Four of the genes that exhibited significant changes were related to anti-viral response and only one to platelet activation (Fig. 3). IFI27 and IFI6 expression remained significantly elevated in COVID-19 patients likely due to an overall increase in the anti-viral response. RSAD and STAT1 also respond to interferon and participate in the innate immune response to viruses. The increased expression of LCP2, an adapter protein for the T cell antigen receptor pathway has been linked to platelet activation in mice. In summary, platelets of patients with severe COVID-19 differ almost exclusively on viral response genes when compared with platelets from patients suffering from non-SARS-CoV-2-related lung inflammatory conditions.

The GO Enrichment analysis indicated that 249 biological processes were significantly enriched in the set of 5 genes (Supplementary Table 7). The number of shared terms between the genes shown in Fig. 3b suggests that IFI27, RSAD2 and IFI6 are tightly connected and take part in several common biological processes whereas STAT1 and LCP2 are separated from the rest.

### Transcriptomic differences between mild/moderate and severe COVID-19 cases

**Figure 4:**
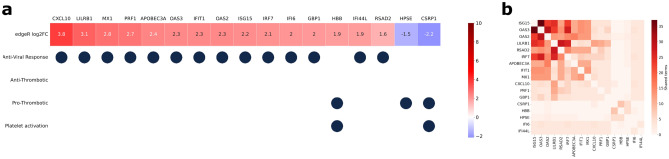
Differences in gene expression between Mild/moderate and Severe and COVID-19 patients for genes related to the selected GO terms. (**a**) GO-term related to each of the differentially expressed genes. The top panel shows the log_2_ fold values for each gene. (**b**) Number of GO-terms shared by each pair of genes among the terms significantly enriched in the ensemble.

Using the available clinical information (Supplementary table 4), we divided the COVID-19 patients into severe (either labeled severe or admitted to ICU) and mild/moderate (either labeled as mild or moderate or admitted to the hospital floor). These two groups presented 66 genes with significant differences in expression (Table 6). Among these genes, we found 14 genes related to anti-viral response processes upregulated in the mild/moderate cases. We also found 3 genes related to thrombosis and platelet activation, one of which (HBB) had higher expression in the mild/moderate cases whereas the other two (HPSE and CSRP1) presented higher expression in the severe cases (Fig. 4a).

The set of differentially expressed genes was enriched in 642 different GO terms (Supplementary table 7). Examining the GO terms shared among the genes revealed two distinct communities, one containing the viral response genes, overexpressed in mild/moderate cases and another one containing the thrombosis and platelet activation genes overexpressed in patients with severe disease (Fig. 4b).

## Discussion

Severe Acute Respiratory Syndrome Coronavirus 2 (SARS-CoV-2), an enveloped single-stranded RNA virus, responsible for COVID-19, predisposes some patients to a proinflammatory and pro thrombotic state^21,22^. Thrombotic and thromboembolic complications are closely linked to mortality^23,24^. Common lab abnormalities in patients suffering from these complications include elevated fibrinogen and D-dimer levels, as well as thrombocytopenia. These are predictors of worse prognosis, intubation and death^25^. Here, we explored the transcriptomic changes present in the platelets from patients infected with SARS-CoV-2 and tried to understand their origin.

Endothelial cell invasion by SARS-CoV-2 leads to cell damage and inflammation, creating a pro-thrombotic state in the arterial and venous vascular beds throughout the body. Whether platelet activation follows the endothelial damage and generalized inflammatory state or are themselves activated by the virus is less clear. In agreement with previous studies^7,8^, we found that platelets from COVID-19 patients present a clearly distinct transcriptomic profile when compared with healthy subjects. Our analysis revealed significant differences in the expression of over 400 genes. These differences are, however, dramatically reduced when COVID-19 patients are compared with subjects suffering from other acute respiratory infections, reducing the total number of differentially expressed to 39. In particular, we found that the only difference in expression related to thrombosis and platelet aggregation between COVID-19 infection and other pathologies was the upregulation of LCP2. This effect can hardly account for the thrombotic complications associated with COVID-19 infections, suggesting that the thrombotic processes do not derive from a COVID-19-specific platelet response. Instead, the increased risk of thrombosis could be a consequence of the cascade triggered by the endothelial damage characteristic of the COVID-19 infection^21,26^. Although patients with severe disease overexpressed genes involved in activation and thrombosis when compared to patients with milder cases, this phenomenon may also be linked to the severity of the inflammatory response.

A receptor for SARS-CoV-2 binding and cell entry is angiotensin converting enzyme-2 (ACE2)^27,28^. Most studies to date on transcriptomic and proteomic analyses of ACE2^6,29^, have failed to detect ACE2 on control and infected platelets and megakaryocytes. However, a more recent study by Barrett et al. showed that SARS-CoV-2 may infect megakaryocytes via an ACE2-indepent infection mechanism^7^. Our observations confirm the absence of ACE2 expression, as no transcripts were found in the platelets of any of the three datasets analyzed here. However, the presence of the virus inside the platelets remains unclear. The single-cell data from Wilk et al.^13^ analyzed here did not contain any RNA reads aligned to the reference genome of the virus; similarly, Barrett et al. identified virions inside platelets via electron microscopy imaging^7^, but the presence of viral RNA was not confirmed. The recent study by Koupena et al. also found negative results when trying to detect the SARS-CoV-2 RNA in platelets from COVID patients, but managed to detect fragments of the viral genome and, most importantly, to observe internalization of the virus *in vitro*^8^. The internalization triggered apoptotic states, in agreement with the transcriptomic analysis done by the same authors and us, which suggests an upregulation of apoptotic and necroptotic pathways. It seems that if virus invasion does occur, it leads cell death, rather than platelet activation or aggregation.

Our results add evidence to the absence of ACE2 expression in platelets, inferring that if viral infection takes place, it is mediated by an ACE2-independent mechanism. The transcriptomic analysis illustrates how the use of inadequate controls can vastly overestimate the changes in expression caused specifically by COVID-19 infection. The contrast between patients with COVID-19 and subjects with other respiratory conditions shows that the virus does not trigger a direct pro-thrombotic response in the platelets but does spark a stronger anti-viral response than in other respiratory conditions. Therefore, we propose that the thrombotic complications associated with COVID-19 are derived from the endothelial damage and the overall inflammatory response and not from a direct platelet activation by the virus.

## Methods

### scRNA-seq data

Data corresponding to the studies from Combes et al.^17^ and Lee et al.^18^ was obtained from the Gene Expression Omnibus public repository (accession numbers **GSE163668** and **GSE149689** respectively). The data from Wilk et al.’s study^13^ was obtained from the COVID-19 Cell Atlas hosted by the Wellcome Sanger Institute^30^. A summary of the number of patients and number of cells present on each dataset is presented in Supplementary Table 4. Although the specific Sars-CoV-2 variants are not specified in the data sources, the timing of their submission is consistent with all cases reported corresponding to the alpha variant.

### Data pre-processing

The datasets from Combes et al. and Lee et al. were subject to a quality control (QC) process aimed at removing technical artifacts. We filtered each of the datasets independently, based on their distributions of counts, genes, and fraction of mitochondrial content (Supplementary Table 5). The dataset from Wilk et al. Was already pre-processed and therefore we did not filter any cells from it.

After the QC, we normalized the counts on each of the datasets using scran^31^.

### Platelet detection

We used the Scanpy toolkit^32^ to detect the 1500 most variable genes on each dataset, perform dimensionality reduction by principal component analysis (PCA) and build a neighborhood graph in the PC space. The cells were then clustered based on the neighborhood graph using the Louvain-Jaccard algorithm^33,34^ and the platelets cluster was identified on each dataset based the expression of PBPP, TUBB1 and LIMS1. The dimensionality reduction and clustering process was performed again on the isolated cells of the selected cluster to refine the cell selection, using the same positive markers and other markers (S100A8, MS4A1, CD14…) to discriminate against other cell types commonly present in peripheral blood datasets.

### Data integration and differential expression

For visualization purposes, we integrated the data using mutual nearest neighbors-batch correction^19^ on the raw counts of each dataset. We performed PCA on the integrated data and projected it into a 2-dimensional space using a uniform manifold approximation and projection (UMAP)^35^.

To perform the differential expression analysis, we relied on the *muscat* R package^36^ to generate pseudo-bulk data from the original (non-normalized) count data of each subject by computing the sum across all their platelet cells. We then applied the edgeR method^37^ to compare the expression levels across different subject groups and used FDR < 0.05 as the significance threshold.

### Biological process enrichment analysis

We compared platelets from COVID-19 patients and healthy subjects, by using the GO Consortium server^38,39^ to perform a GO biological process enrichment analysis on the sets of differentially expressed genes. The analysis was run using the PANTHER Overrepresentation Test^40^ (released 20,210,224) with all the human gene sin the PANTHER database (annotation version GO Ontology database https://doi.org/10.5281/zenodo.5228828, released 2021–08-18). The analysis was performed using a Fisher’s exact test with false discovery rate correction.

On the subsequent comparisons (Figs. 2, 3, and 4), we determined the GO term enrichment in the selected sets of genes using the enrichGO function from the clusterProfiler R package^41^. The parameters were set to use the Biological Process (“BP”) ontologies and to filter the results using a 0.05 FDR threshold.

### Gene selection

We compiled lists of genes related to pro- and anti-thrombosis, platelet activation and anti-viral response using the following gene ontology (GO) terms on the AmiGO database^42^:

- Pro-thrombotic: Platelet aggregation (GO:0,070,527), Positive regulation blood coagulation (GO:0,030,194), Positive regulation of coagulation (GO:0,050,820), Fibrin clot formation (GO:0,072,378).
- Anti-thrombotic: Negative regulation blood coagulation (GO:0,030,195), Negative regulation of coagulation (GO:0,050,819).
- Platelet activation: Platelet activation (GO:0,030,168)
- Anti-viral response: Defense response to virus (GO:0,051,607), Cellular response to virus (GO:0,098,586)

Information on the annotated function of the selected genes was obtained from the GeneCards database^43^.

## Supplementary Information

Supplementary Information 1.

Supplementary Information 2.

Supplementary Information 3.

Supplementary Information 4.

Supplementary Information 5.

Supplementary Information 6.

Supplementary Information 7.
