# Supplementary material for: Integrated analysis of transcriptomic data reveals the platelet response in COVID-19 disease
Source: Sci Rep. 2022 Apr 27;12:6851. doi: 10.1038/s41598-022-10516-1 (PMC9043882; doi:10.1038/s41598-022-10516-1)
Supplement: Supplementary file 4 — Supplementary Information 4. [file 41598_2022_10516_MOESM4_ESM.docx]

|  | COVID-19 subjects | Admission: ICU | Admission: Floor | Severe | Mild | Healthy controls | Other respiratory conditions | Cells |
| --- | --- | --- | --- | --- | --- | --- | --- | --- |
| Wilk et al. | 7 | 6 | 1 | N/A | N/A | 4 |  | 44721 |
| Combes et al. | 21 | N/A | N/A | 10 | 11 | 14 | 11 | 90723 |
| Lee et al. | 11 | N/A | N/A | 6 | 5 | 4 |  | 85144 |

Supplementary Table 4. Number of subjects and cells present on each of the datasets employed in the study.
