# Supplementary material for: Integrated analysis of transcriptomic data reveals the platelet response in COVID-19 disease
Source: Sci Rep. 2022 Apr 27;12:6851. doi: 10.1038/s41598-022-10516-1 (PMC9043882; doi:10.1038/s41598-022-10516-1)
Supplement: Supplementary file 5 — Supplementary Information 5. [file 41598_2022_10516_MOESM5_ESM.docx]

|  | minimum counts per cell | maximum counts per cell | minimum genes per cell | maximum genes per cell | maximum fraction of mitochondrial counts |
| --- | --- | --- | --- | --- | --- |
| Combes et al. | 200 | 30000 | 200 | 5000 | 0.1 |
| Lee et al. | 250 | 50000 | 750 | 6000 | 0.15 |

Supplementary Table 5. Thresholds used during the quality control process for filtering out artifacts on the datasets of Combes et al. and Lee et al. These thresholds were chosen based on the distributions of the different features on each of the datasets.
